# The Coming of Age of Preclinical Models of MDS

**DOI:** 10.3389/fonc.2022.815037

**Published:** 2022-03-16

**Authors:** Wei Liu, Patric Teodorescu, Stephanie Halene, Gabriel Ghiaur

**Affiliations:** ^1^ Section of Hematology, Yale Cancer Center and Department of Internal Medicine, School of Medicine, Yale University, New Haven, CT, United States; ^2^ Department of Oncology, The Johns Hopkins Hospital, Johns Hopkins Medicine, Baltimore, MD, United States

**Keywords:** humanized mouse models, immunodeficient mouse models, transgenic mouse models, xenograft animal model, myelodysplastic syndromes (MDS)

## Abstract

Myelodysplastic syndromes (MDS) are a heterogeneous group of clonal bone-marrow diseases with ineffective hematopoiesis resulting in cytopenias and morphologic dysplasia of hematopoietic cells. MDS carry a wide spectrum of genetic abnormalities, ranging from chromosomal abnormalities such as deletions/additions, to recurrent mutations affecting the spliceosome, epigenetic modifiers, or transcription factors. As opposed to AML, research in MDS has been hindered by the lack of preclinical models that faithfully replicate the complexity of the disease and capture the heterogeneity. The complex molecular landscape of the disease poses a unique challenge when creating transgenic mouse-models. In addition, primary MDS cells are difficult to manipulate *ex vivo* limiting *in vitro* studies and resulting in a paucity of cell lines and patient derived xenograft models. In recent years, progress has been made in the development of both transgenic and xenograft murine models advancing our understanding of individual contributors to MDS pathology as well as the complex primary interplay of genetic and microenvironment aberrations. We here present a comprehensive review of these transgenic and xenograft models for MDS and future directions.

## Introduction

Myelodysplastic syndromes (MDS) are a heterogeneous group of clonal bone-marrow diseases which have in common ineffective hematopoiesis resulting in cytopenias and morphologic dysplasia of hematopoietic cells. MDS is the most common myeloid malignancy in the United States, with a median age at diagnosis of 72 years (1). The diagnosis needs to be supported by the presence of persistent cytopenias (otherwise unexplained) of at least one lineage and morphologic dysplasia of hematopoietic elements or the presence of certain genetic aberrations [del(5q)]. Genetic evidence of clonal hematopoiesis can also contribute to the diagnosis, but as of today, this is not required (Hasserjian, Pathobiology 2019).

The spectrum of genetic abnormalities identified in MDS is wide, ranging from chromosomal abnormalities such as deletions/additions (del(5q), del(7q)), to specific mutations affecting the spliceosome (SF3B1, SRSF2), epigenetic changes (TET2, ASXL1, DNMT3A) or transcription factors (RUNX1, ETV6). While the mechanism by which some of these mutations lead to disease are not fully understood, a number of them have important implications for diagnosis and prognosis (i.e., SF3B1 – ring sideroblasts), and can guide initial treatment (del(5q) – lenalidomide, SF3B1 – luspatercept).

The course of the disease is variable, correlating with the risk-category. Thus, low-risk MDS has an indolent course, characterized by low-grade cytopenias, not requiring treatment for a long time. On the other hand, high-risk MDS is an aggressive disease characterized by profound cytopenias requiring urgent treatment and increased progression to AML. To some extent, high, and low risk MDS appear as two biologically distinct entities. To this end, they also display different combinations of somatic mutations. For instance, SF3B1 are more likely to segregate with low-risk disease (particularly SF3B1K700E), whereas other mutations (i.e., ASXL1, RUNX1, TP53, EZH2, ETV6 and SF3B1K666N) are usually associated in high-risk MDS.

Currently, the therapeutic options in MDS are limited and include supportive care (blood transfusions, antibiotics) and several pharmacologic interventions. Erythropoietin and hypomethylating agents, such as Azacitidine and Decitabine have been the main therapeutic interventions for many years, and immunomodulating agents, such as Lenalidomide are beneficial for patients with del(5q). Most recently, luspatercept (a TGFβ-pathway activin receptor trap) was approved for the treatment of transfusion-dependent MDS with ring-sideroblasts. Nevertheless, none of these treatment options are curative, and in the absence of bone marrow transplantation patients eventually succumb to cytopenias-related complications (infections, hemorrhage) or progression to AML. The exact mechanism by which specific therapeutic interventions interact with downstream consequences of various mutations is currently unknown (2).

As opposed to AML, research in MDS has been hindered by the lack of preclinical models. First of all, the complex molecular landscape of the disease poses a unique challenge when creating transgenic mouse-models. In addition, primary MDS cells are difficult to manipulate *ex vivo* resulting in a paucity of cell lines and patient derived xenograft models. While high-risk MDS models are probably closer to AML models, the aforementioned challenges are especially difficult to overcome when modeling low-risk MDS. However, in recent years, progress has been made to develop both transgenic and xenograft strategies, with some models reproducing the disease more closely than others.

## Mouse Models of MDS

Murine models offer sophisticated tools to dissect unique aspects of mammalian hematopoiesis from phenotype – function relations in various compartments to complex interactions between hematopoietic cells and their microenvironment during ontogeny. More so, breeding strategies and the development of pure genetic backgrounds has allowed the scientific community to isolate and clearly define the impact of genetic alterations on hematopoiesis.

**Figure 1:**
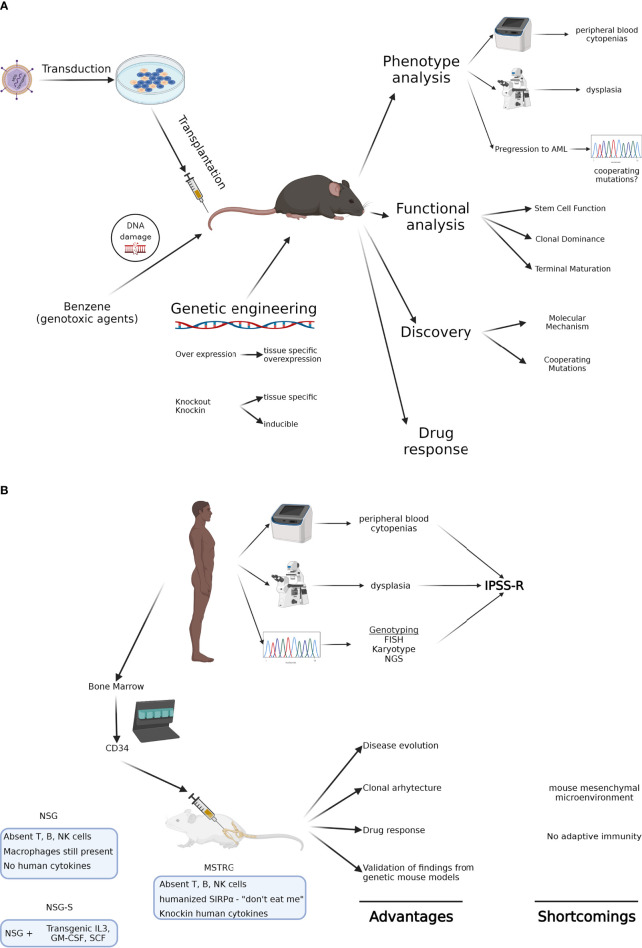
**(A)** Genetic mouse models of MDS. Using various approaches, these animals allow modeling of MDS in immune competent hosts and in the presence of the endogenous, often unmutated microenvironment. They also provide the analytical tools to study how various mutations impact stem cell function and clonal dominance. Their major shortcoming is that they don’t capture the genetic heterogeneity of MDS. **(B)** Xenograft mouse models of MDS. Using patient-derived MDS cells, these animals allow modeling of genetically complex disease and the study of clonal architecture and clonal evolution. Most recent humanized immunodeficient mice can even model erythroid maturation, though limited generation of neutrophils and platelets are thus far a major limitation. Created with BioRender.com.

In 2002 the Hematopathology subcommittee of the Mouse Models of Human Cancers Consortium devised criteria for MDS in mouse models to allow investigators to diagnose lesions as well-defined entities according to universally accepted criteria. Using peripheral blood findings, cytologic features of hematopoietic tissues, histopathology, immunophenotyping, genetic features, and clinical course, they distinguished nonlymphoid leukemias, nonlymphoid hematopoietic sarcomas, myeloid dysplasia, and non-reactive myeloid proliferations (3). These criteria have allowed the uniform evaluation of models and have been particularly useful in mouse models of MDS (**Figure 1A**).

### Informed by Environmental Exposure

Initial efforts to model MDS in mice used exposure to genotoxic agents to create the disease (4–6). These models provided insight into adaptive physiological processes that are deployed in the setting of bone marrow failure from MDS. Even though this strategy resulted in complex genetic diseases, it was not suitable to explore the contribution of individual gene mutations to the development of MDS.

### Informed by Gene Expression Data

Gene expression analysis resulting from microarray and more recently RNA sequencing studies hinted towards recurrent molecular patterns present in patients with MDS. Effort to reproduce these patterns in mice took advantage of either “transduction-transplantation” approaches or transgenic mouse technology to create murine models of MDS. In 2010 Beachy et al. provided a comprehensive review of mouse models engineered to replicate alterations in gene expression identified in patients with MDS, replicating some but not all aspects of MDS (7). These models included combinations of *Pten/Ship* deletions, *Evi1* overexpression, *Npm1* deletion, *Dido* deletion, *Nup98-Hoxd13* fusion, *SALL4B* overexpression, co-expression of *BCL-2* and mutant *NRAS (NrasD12)*, over-expression of mutant *Runx1*, deletion of *Arid4a*, and knock-in of mutant *Polg.* Though an in depth discussion of each of these mice is beyond the scope of this limited review, we will highlight three of these mouse models to capture the diverse biology of MDS.

Overexpression of *Nup98-Hoxd13* fusion under control of the Vav1 promoter generated perhaps one of the most used transgenic mouse models of MDS, NHD13. *NUP98* encodes a component of the nuclear pore complex that mediates nucleo-cytoplasmic transport of RNA and protein. Translocations of this gene have been identified in various hematologic malignancies (MDS, AML, CML, pre-T LBL) and frequently the partner genes encode homeodomain proteins belonging to the group of HOX genes. As a result of expression of the NUP98-HOXD13 fusion gene in all hematopoietic tissues, NHD13 mice developed MDS with peripheral leukopenia, neutropenia, and anemia, while the BM was normo- or hypercellular. More than half of the mice eventually progressed to acute leukemia within 18 months (8).

*SALL4* is a gene encoding a zinc-finger transcription factor and has two isoforms – SALL4A and SALL4B. Its constitutive expression may play a role in AML pathogenesis. Transgenic mice constitutively expressing human SALL4B developed MDS-like features such as increased number of immature blasts, atypical and dysplastic WBCs, with hyper-segmented neutrophils and pseudo-Pelger-Huet-like cells. Dysplasia was also present in the other lineages, with binucleate erythroid precursors and hypo-lobated megakaryocytes, as well as giant platelets. In addition, around 50% of these mice eventually progressed to AML. These changes were attributed to activation of the Wnt/ß-catenin pathway by the constitutively expressed SALL4B (9).

Even though genomic alterations are by far the most studied events in the pathogenesis of MDS, there is increasing interest in understanding if/how epigenetic events may contribute to disease homeostasis. Such events may alter energy metabolism or the microenvironment of hematopoietic cells and thus promote disease initiation and maintenance. For instance, ablation of the proof reading function of DNA polymerase gamma (POLG), which is responsible for the replication of mitochondrial (mt)DNA, resulted in mtDNA mutations. As a result, *Polg* deleted mice showed various features of accelerated aging. Starting at the age of 6 months, mutant mice showed progressive macrocytic anemia suggestive of MDS, which worsened rapidly after the age of 11 months. In addition, both B and T lymphocyte counts were decreased. However, none of these mice progressed to acute leukemia (10).

### Informed by Recurrent Mutations

Over the last 15 years, there has been clear evidence that genetic mutations in several conserved pathways are frequent in MDS and amenable to modeling in mice (**Table 1**). Of these, mutations in transcription factors (e.g. RUNX1, ETV6, BCOR), epigenetic modifiers (DNMT3A, TET2, EZH2, ASXL1), and most recently splicing factors (SRSF2, U2AF1, SF3B1 and ZRSR2) have been already reported in mouse models that offer insight into MDS biology. Mutations in genes belonging to these categories alone are generally insufficient to reproduce all clinical and biological features of human MDS. These models and the mechanistic understanding they have provided now offer the opportunity to recreate the genetic heterogeneity and understand the mutations’ synergy in MDS either in silico or by generating models that combine genetic mutations that frequently co-occur in patients.

**Table 1:** Mouse models of MDS.

Genetic aberrations	Techniques	Reference
***Gene expression***		
*Pten/Ship* deletions	*Pten* haploinsufficient, *Ship* knockout	(11)
*Evi1* overexpression	Retroviral transduction	(12)
*Npm1* deletion	*Npm1* haploinsufficient	(13, 14)
*Dido* deletion	Knockout	(15)
*NUP98-HOXD13* fusion	Transgenic expression (*Vav* promoter)	(16–19)
*SALL4B*	Transgenic expression (CMV promoter)	(9)
*Bcl2*/mutant *Nras*	Transgenic co-expression (*tTA, MRP8* promoters)	(20)
*Arid4a*	Knockout	(21)
*Polg*	Knock-in of mutant (Polg^A/A^)	(10)
MLL fusions	Retroviral transduction	(22)
***Recurrent mutations***		
**Transcription factors**		
*RUNX1*	Retroviral expression (D171N, S291fs)	(23)
*Bcor*	Loss-of-function mutation	(24)
**Epigenetic modifiers**		
*Dnmt3a*	*Mx1-Cre* mediated ablation	(25)
*Tet2*	*Tet2:nlacZ/nGFP* knockin, which results in nlacZ transcription	(25)
*Ezh2*	*Rosa26:Cre-ERT* mediated ablation, followed by HSPC transplant	(26)
*Axsl1*	Homozygous/heterozygous *Asxl1:nlacZ/nGFP* knockin	(27)
**Splicing factors**		
*Srsf2*	*Mx1-Cre* mediated heterozygous expression of *Srsf2P95H*	(28)
*U2af1*	Doxycycline inducible heterozygous expression of *U2af1S34F*	(29)
*Sf3b1*	*Mx1-Cre* mediated heterozygous expression of *Sf3b1K700E*	(30)
*Zrsr2*	*Mx1-Cre* mediated ablation	(28)
**Chromosomal aberrations**		
del(5q)	Deletion of *Cd74-Nid67* interval	(31)
**BMME dysfunctions**		
*Dicer1*	*Osx-Cre* mediated ablation	(32)
*Sbds*	*Osx-Cre* mediated deletion	(32, 33)
*S100A9*	Transgenic overexpression	(34)

#### Transcription Factors

*RUNX1* or *AML1* encodes a transcription factor with location on chromosome 21q22 and is the most frequent target for chromosomal translocation in leukemia. Mice transplanted with bone-marrow cells infected with a retroviral vector harboring mutant RUNX1 developed MDS-RAEB or MDS/AML with high penetrance. Two types of mutations were used – one located in the Runt homology domain (RUNX1^D171N^) and the other one causing a frameshift leading to a C-terminal truncation (RUNX1^S291fs^). While the first one led to leukocytosis and hepatosplenomegaly in mice, the latter caused leukopenia. Interestingly, in both cases multilineage dysplasia was present, with Howell-Jolly bodies, red cell polychromasia, and poikilocytosis. Giant erythroblasts, karyorrhexis, and macrocytosis were also detected. Pseudo-Pelger-Huët anomaly, hyper-segmented neutrophils, and giant platelets were also seen in mice harboring the RUNX1^D171N^ mutation. As a result, MDS/AML was diagnosed in the vast majority of mice in both groups, while a small number of animals classified as MDS-RAEB. The differences between the 2 phenotypes are likely due to the higher expression of Evi1 found only in the RUNX1^D171N^ mice. This is supported by the fact that co-transduction of BM cells with Evi1 and RUNX1^D171N^, but not RUNX1^S291fs^, rapidly reproduced the phenotype seen in the transgenic models (23).

*EVI1* is a proto-oncogene that has been associated with hematologic malignancies in both mice and men. The transgenic Evi1 mouse showed defects in erythroid hematopoiesis with a reduction in CFU-E derived colonies, but without differences in CFU-G, CFU-GM and CFU-MK derived colonies. More so, there were no other phenotypes observed in the peripheral blood, bone marrow, and spleen of these mice. In the transgenic line harboring the highest number of transgene copies, the phenotype was more severe and showed an important reduction in spleen size, complete absence of the red pulp and erythroblasts, and neutrophil infiltration. Neutrophil infiltration and reduction of erythroblasts were also seen in the bone marrow of these mice. Although reticulocyte counts were decreased, the number of circulating RBCs was normal. Interestingly, in this transgenic line, the Evi1 transgene is X-linked, thus the described phenotype is only present in males (35).

#### Epigenetic Modifiers

Up to 30% of patients with MDS show alterations in the Ten-Eleven-Translocation-2 (TET2) gene, which is also involved in other myeloid malignancies, such as MPN, CMML, and AML. The TET gene family epigenetically regulates gene expression by opposing methylation-driven gene silencing, thus possibly acting as a tumor suppressor gene. Several genetically engineered Tet2^-/-^ mice models were generated revealing that *Tet2* deletion was sufficient to initiate myeloid and lymphoid malignancies in mice (36–39). They developed leukocytosis with monocytosis and neutrophilia as early as two months of age. During aging, the BM, spleen and liver became infiltrated with erythroblasts and mature myeloid cells. Based on the Bethesda criteria, the phenotype was heterogeneous and best defined as MDS with erythroid predominance, CMML, or myeloid leukemia with maturation (37). They showed hematopoietic stem cell expansion and myeloid and lymphoid transformation (36, 38, 39).

Mice deleted for another epigenetic modifier gene, *Dnmt3a*, revealed an aberrant phenotype affecting all hematopoietic cell lineages. DNMT3A is a methyltransferase frequently mutated in myeloid malignancies such as MPN, MDS and AML. The knockout mice displayed marked myeloid and erythroid dysplasia in their peripheral blood with increased myeloid cells. Bone marrow cellularity was also increased displaying multilineage dysplasia with impaired erythroid maturation. Spleen and liver showed myeloid infiltration with increased blasts, dysplastic megakaryopoiesis, and erythrophagocytosis. These findings were consistent with a diagnosis of MDS/MPN with extramedullary hematopoiesis (40).

ASXL1, a member of the Polycomb group, is altered in various myeloid malignancies (MDS, MPN, CMML, JMML, AML) and generally associated with worse prognosis. The knockout (KO) of both alleles of the gene in mice led to severe developmental abnormalities, such as dwarfism and anophthalmia, and an 80% embryonic lethality. The few surviving mice exhibited multiple cytopenias and dysplastic features such as presence of hyper- and hypo-segmented neutrophils, pseudo-Pelger-Huët anomaly, increased numbers of polychromatophilic RBCs and Howell-Jolly bodies. The BM of these mice was normo- or hypercellular, with myeloid hyperplasia and erythroid hypoplasia, and micromegakaryocytes with hypolobated nuclei. Furthermore, the spleens were small due to reduced red pulp and smaller lymphoid aggregates in the white pulp. As the majority of *ASXL1* mutations in patients are heterozygous, *Asxl1* haploinsufficient mice were also developed. Heterozygous KO mice recapitulated the phenotype and also showed hyper- and hypo-segmented neutrophils, pseudo-Pelger-Huët anomaly, frequent apoptotic, and hypogranulated neutrophils and increased polychromatophilic RBCs. The spleen architecture was also disrupted, and the BM showed an increased proportion of myeloid cells and a decrease in erythroid islands. The phenotype was more pronounced with age with some mice developing profound anemia, thrombocytopenia, leukopenia, and in some cases leukocytosis and monocytosis, suggesting disease progression with aging (27).

#### Splicing Factors

After the discovery of recurrent mutations in key factors of the splicing machinery in greater than 50% of patients with MDS and in a subset of patients with MDS/MPN overlap syndromes such as CMML and AML in *SRSF2*, *U2AF1*, *SF3B1* and *ZRSR2* by Yoshida et al. and others (41, 42), several groups generated inducible knockin [*Srsf2* (28, 43), *Sf3b1* (30, 44, 45)], transgenic [*U2af1* (29, 46)], or knockout [*Zrsr2* (47)] mouse models. These mouse models replicated the most common mutations identified in patients. All models phenocopied aspects of MDS but to varying degrees. One important question raised was whether induction of mutations provided mutant stem cells with a competitive advantage as seen in patients in whom splicing factor mutations are almost always a part of the dominant clone. Surprisingly, most models instead showed a competitive disadvantage (Kim, Obeng, Shirai, Mupo, Seiler) for long-term hematopoietic stem cells (LT-HSCs) even though some showed robust initial engraftment when mutant cells were engrafted into irradiated recipient mice to prove the cell-intrinsic nature of the defect (44). Analysis of expression of the mutant versus the wildtype splicing factor transcript in the obligatory heterozygous models revealed expression levels closer to 30%, dependent on the technology used to derive these inducible models. A second *Srsf2* P95H mutant model generated in 2018 by the Walkley group achieved expression of mutant *Srsf2* closer to 50% as found in patients and exhibited the selective advantage of mutant HSCs expected in MDS. For comprehensive review of the *U2af1*, *Srsf2*, and *Sf3b1* mutant mouse models we refer the reader to a comprehensive review (48). Interestingly, both *Srsf2* mutant and *Zrsr2* knockout mouse models showed stronger phenotypes resembling MDS than *U2af1* or *Sf3b1* mutant mice, which could be attributed to the binding preferences of SRSF2 and ZRSR2. Exons bound by SRSF2 and introns bounds by ZRSR2 as part of the minor (or U12) spliceosome (49) are more highly preserved between human and mouse (50–52) than intronic sequences bound by U2AF1 or SF3B1, resulting in higher overlap of specific genes affected by the respective mutant splicing factors.

#### Compound Mutant Mouse Models

Since generation of single mutant mice, compound mutant mice have been generated, providing better understanding of the progression from clonal hematopoiesis to MDS and to AML. Examples include SRSF2/IDH2 co-mutant mice that exhibited profound myelodysplasia and rapid progression to AML likely *via* reduced expression of INTS3, a member of the integrator complex (53). Similarly, NHD13 MDS mice exhibited accelerated progression to acute leukemia when combined with the Vav1 driven IDH2^R140Q^ transgene, though the phenotype of their leukemia resembled early T-cell precursor ALL rather than AML (54). In contrast, SRSF2/TP53 co-mutant mice did not show increased progression to AML (43) even though loss of TP53 accelerated progression to AML of NHD13 MDS mice (55). On the other hand, U2AF1/RUNX1 co-mutant mice showed normal survival and did not progress to AML unless exposed to alkylating agents (56).

#### Chromosomal Aberrations

The deletion of the long arm of chromosome 5 [del(5q)] is the most common karyotype abnormality in *de novo* MDS and defines its own subtype of MDS. While the underlying mechanism of pathogenesis is not completely understood, the loss of several genes located within the deleted region has been identified as possible disease initiating events. Among these, RPS14 (a ribosomal protein) (57), CSNK1A1 (a serine/threonine kinase) (58), and two microRNAs – miR-145 and miR-146a (59) are likely to play a role in the phenotype of del(5q) MDS. In mice, the genes equivalent to the ones located in the 5q region in humans, are located on chromosome 18. Therefore, a mouse model was generated with a deletion of the cd74-nid67 region on chromosome 18 to better understand the pathogenesis of the human 5q- syndrome. These mice developed pronounced macrocytic anemia, thrombocytopenia, and granulocytopenia. They also had a hypocellular bone marrow with a deficit in the hematopoietic progenitor populations (31). Further analysis of the bone marrow compartment showed an accumulation of TP53 protein, cell cycle arrest and increased apoptosis. However, when crossed with TP53^-/-^ mice, the phenotype was almost completely reversed, except for the low RBC counts (present in the original TP53^-/-^ mice as well) and the macrocytosis. More so, activation of the TP53 pathway was associated with loss of RPS14 and increased ribosomal stress (60).

#### Bone Marrow Microenvironment (BMME) Dysfunction in MDS Pathogenesis

The bone-marrow microenvironment was shown to play an important role in different stages of MDS. Although its most prominent contribution is probably in disease maintenance and progression, several studies have shown that deleting particular genes in the microenvironment can actually initiate MDS.

Dicer1 is an RNase III endonuclease involved in the processing of RNA and in microRNA biogenesis. The deletion of this gene in mouse osteoprogenitor cells in the Osx-GFP-Cre^+^Dicer1^fl/fl^ mouse model resulted in impaired osteoblastic differentiation and decreased calcified matrix deposition (61). This alteration of the bone marrow niche led to myelodysplasia in these mice. Leukopenia was present in all cases, while some animals also displayed profound anemia and thrombocytopenia. While the BM was normo- or hyper-cellular, no differences were found in the hematopoietic stem and progenitor cells. However, bone marrow of these mice showed dysplastic features such as hyper-segmented nuclei in neutrophils, giant platelets, and micro-megakaryocytes with hypo-lobulated, hyperchromatic nuclei. Consistent with human MDS, B-cells and B-cell progenitors were reduced in the BM in favor of an increased frequency of myeloid cells. In addition, a small percentage of these mice progressed to either AML or myeloid sarcomas. The essential role of the microenvironment in disease initiation in this model was highlighted by reciprocal transplantation experiments in which the disease could not be reproduced in wild type recipient mice. Microarray analysis of the transcriptome of Dicer^-/-^ osteolineage cells suggested that a significant downregulation of the Schwachman-Diamond-Bodian syndrome gene (SBDS) might be responsible for the observed phenotype (32). This hypothesis was further strengthened by the fact that deletion of the SBDS gene in the same compartment in mice led to a similar phenotype by activating the p53-S100A8/9-TLR inflammatory signaling axis, thus driving genotoxic stress (33).

The role of the bone marrow microenvironment in MDS maintenance was also clearly demonstrated in NHD13 mouse models. Even though transgene expression is restricted to hematopoietic cells *via* the Vav1 promoter-enhancer, these mice showed characteristic MDS-induced alteration of the bone marrow microenvironment including increased endothelial cells and dysfunctional mesenchymal and osteoblastic cellular populations (62, 63). They are, thus, a model to study the interactions between the mutant hematopoietic clone and the surrounding microenvironment and the role of various chemo-/cytokines in the MDS phenotype.

## Xenograft Models of MDS - Why Are Patient Derived Xenograft (PDX) Models Needed?

### Inherent Differences Between Mice and Humans

Although the mouse models described above present with several phenotypic features of MDS and allow for in depth characterization of gene function and characterization of mutations, they still have obvious limitations with respect to their ability to recapitulate human MDS. While many gene functions are preserved between mammalian species, some are not and their targets may differ greatly when comparing human and murine species. This has for example become particularly obvious in murine models of splicing factor mutations, affecting 50% of patients with MDS; hallmarks of splicing factor mutant MDS, such as ring sideroblasts in SF3B1 mutant MDS, are absent in *Sf3b1* mutant mice (30). Even in monogenic bone marrow failure disorders such as Fanconi Anemia, mouse models fail to recapitulate the human phenotype (64–66). In addition, murine models generally do not replicate genetic complexity or clonal evolution encountered in patients, especially under treatment pressures, making it imperative to study these diseases in primary human cells (**Figure 1B**).

#### Limited Availability of MDS Cell Lines

The study of primary human MDS poses a particular challenge that continues despite several critical improvements over the past several years. The MDS hematopoietic stem cell is defective, reminiscent of stem cell dysfunction in the inherited bone marrow failure syndromes such as Fanconi Anemia. While a few human cell lines have been successfully generated from patients whose bone marrow failure has transformed to leukemia, cell lines that recapitulate the bone marrow failure state are rare and limited in their genetic diversity [reviewed in (67)]. Drexler et al. reviewed 31 candidate MDS cell lines and classified them into three categories: (1) false (cross-contaminated) cell lines and non-malignant cell lines; (2) malignant cell lines established at the AML/MDS leukemic phase but not MDS phase; and (3) MDS cell lines established during the MDS phase (67). Among these cell lines, three cell lines were established during the MDS phase of the diseases. In 1991, the MDS92 cell line was derived from the bone marrow of a 52-year-old male with RARS which developed into RAEB, but prior to leukemic transformation. This cell line carries a complex karyotype, including 5q- and -7, as well as a codon 12 mutation in NRAS. The MDS92 cell line is cytokine-dependent (68, 69); a blastic MDS-L subline was derived in 2000 (70) and shown to be responsive to lenalidomide (71). In 1994, the M-TAT cell line was established from the peripheral blood of a 3-year-old male at relapse of RAEB-T. This cell line is also cytokine-dependent for growth and responds to various cytokines with differentiation down the erythroid or megakaryocytic lineages (72). The TER-3 cell line was established in 2002 from a male patient’s bone marrow at time of progression from RA to RAEB. The complex karyotype included monosomies 7 and 20 among other aberrations. Like M-TAT, this cell line is constitutively cytokine-dependent with potential to differentiate towards the erythroid and megakaryocytic lineages (73).

#### Immunodeficient Mouse Models

Given the limited growth potential of primary MDS cells *in vitro* and the limited number and fidelity of MDS cell lines, models are necessary that allow propagation of primary, patient- derived MDS to allow study of the highly heterogeneous disease. The immunodeficient murine host has become the ideal host to study human tumors *in vivo*, from an ethical, practical, and cost standpoint.

Historically, direct transplantation of human cells into immune competent mice failed to give positive outgrowth due to immune rejection by the recipient mice; sublethally irradiated mice died without reconstitution of hematopoiesis (74). Since then immunodeficient mouse models have undergone a long evolution from a host lacking murine T- and B-cells to multi-lineage immune-deficient models (T-, B- and NK-cells) with additional modifications; identification and introduction of mutations that enhance recognition of self across the murine-human barrier (SIRPalpha); adaptation of the murine host to express human proteins essential for human cell survival and differentiation, such as cytokines; and introduction of human cellular systems, such as mesenchymal stromal cells and entire ossicles. A detailed review of these mouse models is beyond the scope of this review and provided in detail by Martinov et al. (75). Here we seek to provide a brief summary as it pertains specifically to myeloid malignancies and in particular MDS (**Table 2**).

**Table 2:** PDX mouse models of MDS.

Name	Dysplastic lineages	Cytopenias*	Ring sideroblasts as % of marrow erythroid elements	BM and PB blasts	Cytogenetics by conventional karyotype analysis	Engraftment Model
MDS with single lineage dysplasia (MDS-SLD)	1	1 or 2	<15% or <5% if SF3B1 mutant	BM <5%, PB <1%, no Auer rods	Any, unless fulfills all criteria for MDS with isolated del(5q)	MISTRG (76); NSG/NSG-S (77); NSG (78); NOG (79)
MDS with multilineage dysplasia (MDS-MLD)	2 or 3	1-3	<15% or <5% if SF3B1 mutant	BM <5%, PB <1%, no Auer rods	Any, unless fulfills all criteria for MDS with isolated del(5q)	MISTRG (76); NSG/NSG-S (77, 80); NSG (78)
**MDS with ring sideroblasts (MDS-RS)**						
MDS-RS with single lineage dysplasia (MDS-RS-SLD)	1	1 or 2	≥15% or ≥5% if SF3B1 mutant	BM <5%, PB <1%, no Auer rods	Any, unless fulfills all criteria for MDS with isolated del(5q)	MISTRG (76); NSG/NSG-S (77, 80); NSG (78, 81)
MDS-RS with multilineage dysplasia (MDS-RS-MLD)	2 or 3	1-3	≥15% or ≥5% if SF3B1 mutant	BM <5%, PB <1%, no Auer rods	Any, unless fulfills all criteria for MDS with isolated del(5q)	MISTRG (76); NSG/NSG-S (77, 80)
MDS with isolated del(5q)	1-3	1-2	None or any	BM <5%, PB <1%, no Auer rods	del(5q) alone or with 1 additional abnormality except −7 or del(7q)	MISTRG (76); NSG/NSG-S (77, 80)
**MDS with excess blasts (MDS-EB)**						
MDS-EB-1	0-3	1-3	None or any	BM 5%-9% or PB 2%-4%, no Auer rods	Any	MISTRG (76); NSG/NSG-S (77, 80); NSG (78, 81); NOG (79)
MDS-EB-2	0-3	1-3	None or any	BM 10%-19% or PB 5%-19% or Auer rods	Any	MISTRG (76); NSG/NSG-S (77); NSG (78); NOG (79)
**MDS, unclassifiable (MDS-U)**						NSG (78); NSG/NSG-S (80)
with 1% blood blasts	1-3	1-3	None or any	BM <5%, PB = 1% on 2 separate occasions; no Auer rods	Any	
with single lineage dysplasia and pancytopenia	1	3	None or any	BM <5%, PB <1%, no Auer rods	Any	
based on defining cytogenetic abnormality	0	1-3	<15%	BM <5%, PB <1%, no Auer rods	MDS-defining abnormality	
Refractory cytopenia of childhood	1-3	1-3	None	BM <5%, PB <2%	Any	

*Cytopenias defined as: hemoglobin, <10 g/dL; platelet count, <100 × 109/L; and absolute neutrophil count, <1.8 × 109/L. Rarely, MDS may present with mild anemia or thrombocytopenia above these levels. PB monocytes must be <1 × 109/L.

Song et al. performed targeted exome sequencing to validate engraftment of clonal MDS.

Pang et al. and Muguruma et al. performed FISH for monosomy 7 to validate engraftment of clonal MDS.

Muguruma et al. and Meydouf et al. used autologous or allogenetic MSC to enhance MDS engraftment.

#### Abrogation of Murine Adaptive Immunity

In the late 1970s the first attempt at PDX models of human acute myeloid leukemia (AML) employed subcutaneous implantation of patient AML cells into thymectomized, irradiated mice. In these T-cell deficient, B-cell competent mice, AML cells could be grown as discrete tumors under the skin. However the tumors started regressing 6 days after inoculation without lasting tumor cells (82). Over the next decade two physiologically more relevant models were the *bnx* mouse which was generated *via* combination of three mutations, *beige*, *nude* and *xid*, resulting in deficiency of T-, NK- and so-called lymphokine activated killer cells (83), and the severe combined immunodeficiency (*SCID*) mouse that carries a single point mutation in the *protein kinase DNA-activated catalytic polypeptide* (*Prkdc*) gene with impaired T- and B-lymphocyte development but intact NK-cell function and innate immunity (84, 85). While normal human hematopoietic cells (86) and acute lymphoblastic leukemia (ALL) cells (87) could be successfully transplanted into *bnx* or SCID mice (88), primary AML cells still failed to reliably engraft in either mouse model (89). Interestingly, if mice were treated with the cytokine granulocyte-macrophage stimulating factor (GM-CSF) and human mast cell growth factor (MGF) human undifferentiated blast cells were identifiable in the murine bone marrow (90). These studies suggested that remnant immunity in the murine host compromised lasting engraftment of human myeloid cells and that the murine environment lacked factors relevant to human cell survival and proliferation. However, these changes were not introduced until 2003.

In the following years, mouse models that combined T-, B- and NK-cell deficiency, such as the nonobese diabetic (NOD)-severe combined immunodeficiency (NOD/SCID) mice became the main strain used for xenograft studies and supported engraftment of ALL and a subset of AMLs (91–94). Discovery that a polymorphism in the *Sirpa* gene in the NOD mouse strain encodes a variant of the SIRPa receptor that cross-reacts with the human CD47 ligand in part explained the improved engraftment levels. Human cells can engage the CD47/SIRPa “don’t eat me” signal in NOD mice and are thereby at least partially protected from phagocytosis by the murine host macrophages.

In regards to primary human MDS cells, in 2002, Nilsson et al. injected 5q- deficient hematopoietic cells obtained from seven MDS patients into NOD/SCID mice. Mice engrafted with cells from one of these patients showed 12% human (CD45+) engraftment and CD45+CD15+ cells proved to carry deletion of 5q. The mice did not exhibit symptoms and engraftment from 6 additional patients were unsuccessful (95). In another study, Benito et al. transplanted bone marrow cells from MDS patients into sub-lethally irradiated NOD/SCID mice. Human CD45+ cells were present in bone marrow and spleen of these mice, albeit with significant delay when compared with healthy donor cell engraftment. Of note, no clonal precursors were found in these grafts validating the poor reconstitution of MDS HSC derived hematopoiesis compared to remnant healthy HSC derived hematopoiesis in the NOD/SCID environment (96).

#### Abrogation of Murine Innate Immunity

Despite the improvement afforded by the *Nod/Scid* model, it became clear that a more permissible host had to be identified to allow MDS HSCs to engraft. One approach was to eliminate residual NK cell activity in the NOD/SCID mouse by elimination or inactivation of the β2-microglobulin (β2m) gene (97–99) or by antibody-mediated elimination of NK cells (100, 101). Indeed, Thanopoulou et al. (102) reported that MDS cells from 4 of 7 MDS patients that included all MDS subtypes engrafted in NOD/SCID β2-microglobulin-deficient mice (NOD/SCID-β2m^null^). These grafts also taught the scientific community about the biology of MDS: in contrast to healthy donor transplants that first give rise to erythroid lineage predominance followed by dominance of lymphoid engraftment, most MDS transplants were myeloid predominant. Importantly, in 4 out of 4 MDS cases, the reconstituted cells carried the same cytogenetic abnormalities, namely trisomy 8 and 5q-, as the original samples. While these steps represented great advances, overall engraftment levels remained low, < 1%. Another methodology to abrogate NK cells in the *Nod/Scid* background was to delete the IL2 receptor common gamma chain (IL2rγ-/-) resulting in the since widely used NOD-scid-IL2rγ-/- (NSG) mice (103). Despite these improvements, engraftment of low risk MDS in NSG mice remained largely unsuccessful, while human AML samples showed robust engraftment (104).

#### Expression of Human Cytokines in the Murine Host

Clearly, combined abrogation of adaptive and partially innate immunity proved insufficient for MDS engraftment suggesting that signals that could support human hematopoiesis were missing. Feurig-Buske et al. compared MDS engraftment in NOD/SCID-β2m^null^ mice to NOD/SCID-β2m^-/-^ mice expressing human cytokines, specifically human interleukin-3 (IL-3), GM-CSF and Steel factor (SF) (NOD/SCID-β2m^null^-3/GM/SF) from a transgene that previously provided excellent engraftment to AML (Feurig-Buske M, et al., Leukemia 2003). Cytokine expression led to reconstitution from all 7 MDS samples tested but with long-term engraftment documented only from 2. Constitutive expression of huIL3, huGM-CSF and huSCF, improved engraftment of primary human AML (105) and enhanced normal human myelopoiesis (106). Nevertheless, engraftment of MDS remained a challenge. In fact, of the MDS cell lines described above, only the MDS-L subline has been engrafted in immunodeficient mice, specifically in NSGS mice (NSG transgenic expressing huIL3, huGM-CSF and huSCF) (107) and employed in drug testing (108–110). While some groups provided evidence that NSGS mice (~5-35%) consistently showed higher engraftment of MDS than NSG mice (~1-9%) (80) several groups have since confirmed that while transient engraftment is enhanced, the constitutive nature of expression of these cytokines is detrimental to normal and MDS HSCs (111, 112).

#### Reconstitution of the Human Niche

As an alternative to transgenic cytokine expression, several groups tested co-transplantation of mesenchymal stromal cells (MSCs), either derived from immortalized cell lines or from autologous or allogeneic BM derived MSC cultures. Such studies showed mixed results. In one study cells from 6 MDS patients were transplanted into NOD/SCID-β2m^null^ mice along with human stroma-derived cell lines HS5 and HS27a with improved but low engraftment levels (0.71-4.44%) (113). Bone marrow CD34 cells from six patient were intrafemorally injected into NOG mice along with human MSCs, resulting in human CD45+ percentages in mouse BM between ~2% and 89% interestingly at the expense of murine hematopoiesis (79). In another study co-injection of autologous MSCs improved engraftment in NSG mice from 1/7 patient samples to 14/20 patient samples with engraftment levels ranging from 1%-22% (80). Interestingly, MSCs were detectable only about a week post transplantation suggesting that their effect on MDS HSC engraftment was transient. The exact mechanism by which MSCs support MDS HSC engraftment remains to be determined; an MSC effect could not be confirmed in several carefully performed studies in NSG or NSGS mice (77, 114).

The most recently advanced strategy to add human microenvironment for human HSPCs engraftment is using human bone organoids (ossicles). Ossicles are created by seeding of human BM-derived MSCs onto a 3D scaffold composed of extracellular matrix (115) or generated by co-injection of MSCs and matrix material under mouse skin; they have provided a favorable niche for normal and leukemic cells (116).

#### Conditioning of the Niche

Routinely stem cell engraftment requires elimination of cells occupying the stem and progenitor cell niche. This is typically achieved *via* irradiation with doses adjusted to the host’s tolerability. Alternatives include conditioning with busulfan (117, 118), attractive in mice with the *scid* mutation that confers a DNA repair defect that sensitizes host tissues to irradiation. Other alternatives that can be cost-prohibitive include administration of antibody-drug conjugates (119). An attractive option would be a murine host that carries a HSPC defect that would allow normal hematopoiesis at steady state but a competitive disadvantage when challenged with human cells. Indeed, mice bearing mutations in the receptor tyrosine kinase Kit (*Kit^W/Wv^*), the stem cell factor receptor required for normal hematopoiesis, allow long-term engraftment of injected wildtype HSPCs without conditioning (120). When introduced into immunodeficient hosts (*Rag2^−/−^γc^−/−^)* various mutations in *Kit* allow engraftment without irradiation (121) and engraftment across the human-mouse barrier (122). Strikingly, these mice displayed robust reconstitution of human erythropoiesis and thrombopoiesis with terminal maturation in the bone marrow (123). Nevertheless, introduction of the *Kit^W41/W41^* mutation into NSG mice was insufficient to allow robust MDS engraftment (124).

#### Physiologic Expression of Critical Human Cytokines

Abrogation of murine T-, B-, and NK cells *via* abrogation of IL-2Rγ (Il2rg gene deletion or truncation), abrogation of V(D)J recombination *via* the Prkdc^scid^ mutation, or deletion of recombination activating genes (RAG)-1 or RAG-2 (125, 126) in combination with either the *Sirpa* polymorphism encountered in the NOD strain or *via* introduction of the human SIRPA gene results in optimal immunosuppression. Transgenic expression of critical cytokines provided at least transient engraftment of MDS HSCs. In 2014 Rongvaux et al. presented a novel mouse model with knockin of critical cytokines that lacked cross-reactivity between human and mouse (127). Knockin provided two advantages: 1) expression of human cytokines was regulated by the endogenous murine regulatory elements resulting in physiologic expression of human cytokines and 2) deletion of the murine cytokine provided a competitive disadvantage for the murine host HSPCs, “opening” up the niche for human xenografted cells. “MISTRG” mice, so named for the cytokines replaced and the immunodeficient background strain express human macrophage colony-stimulating factor (M-CSF), IL-3 and GM-CSF, and thrombopoietin (THPO) in the Rag2^−/−^, IL2Rγ ^−/−^ background. To provide phagocytic cross-tolerance human signal regulatory protein alpha (SIRPα) was introduced as transgene (127) and later also knocked in (128). MISTRG mice have proved to be the most promising host for engraftment of MDS patient samples to date (76). Cells from all MDS risk groups efficiently engrafted in this strain; engraftment levels were significantly higher especially for low-risk MDS. Unlike in NSG mice, increasing numbers of CD34+ cells resulted in increasing engraftment levels while engraftment levels in NSG mice remained low, suggesting a lower threshold for engraftment in MISTRG mice. While in NSG mice engraftment in female recipients was up to 11-fold higher than in male recipients (129), engraftment in MISTRG mice was not affected by the sex of the recipient mice. CD34+ cells from MDS bone marrow produced myeloid predominant grafts that engrafted long-term and were transplantable into secondary recipients. Additionally, engrafted MDS cells in MISTRG mice gave rise to erythroid and megakaryocytic lineages and replicated MDS heterogeneity, myeloid dysplasia, and clonal complexity and evolution. MDS PDXs replicated drug treatment responses such as cellular differentiation in response to treatment with inhibitors of mutant IDH2 (76). A shortcoming of MISTRG as for other models was the absence of mature neutrophils, red blood cells, and platelets in circulation, limiting full assessment of the myeloid lineage maturation. This was overcome by humanization of the murine liver, resulting in abrogation of murine complement expression and enhanced red cell survival in circulation (130).

In summary, immunodeficient mouse models have undergone rapid evolution over the past decade from bi-lineage immunodeficiency to an intricate combination of adaptive and innate immune tolerance. In addition, humanization of cytokines and growth factors that are critical for human hematopoietic stem and progenitor cell survival and differentiation have transformed the murine environment into one favoring human cell engraftment.

## Future Directions and Current Overall Limitations

Over the last decade, we have seen significant therapeutic advances in AML resulting in a number of drugs being either FDA approved or very advanced in their clinical development. Unfortunately, drug development in MDS is still lagging behind. This may be due to unique biological features of MDS compared to AML, particularly the more complex mutational architecture and perhaps higher interdependency between the dysplastic clones and their surrounding immune and stromal microenvironment. Nevertheless, the lack of preclinical models of MDS compared to AML certainly contributed to the slow start of drug development efforts in this disease. Fortunately, the increased accessibility of transgenic technologies coupled with our improved xenograft tools have closed the gap in providing the much needed preclinical models of MDS. While we have come a long way, some challenges remain.

In regard to the transgenic mouse models, we are just beginning to explore the cell intrinsic interactions between various mutations found in patients with MDS. Given the genetic complexity of this disease, modeling all potential interactions is a daunting task. Could information provided by the phenotype of single mutations be integrated to generate algorithms that predict how the combination of mutations would behave? Even if successful, the cell extrinsic interactions between various mutant clones, these clones and residual “unmutated” hematopoiesis or the “unmutated” immune and mesenchymal microenvironment may not be accurately addressed by the current transgenic mouse models. It is important to remember that even if “unmutated”, the residual hematopoiesis and the immune and stromal microenvironment of patients with MDS are not wild type. Can we use transgenic mice to model these interactions?

In regard to xenograft models, they all rely on hosts that have no adaptive and partially compromised innate immunity and once engrafted with human cells represent xenogeneic immune chimera. Thus, at this point, it remains impossible to study the role of immune senescence for instance in MDS homeostasis. Could adaptive transfer of human B and T cells into some of the most advanced xenograft models (i.e. MISTRG) model these interactions, even for a short period of time? Cytopenias are the root cause of much of the morbidity and mortality and lack of quality of life experienced in MDS. We have made significant progress towards generating xenograft models that allow full maturation of several human hematopoietic cell lineages. Nevertheless, these models continue to imperfectly recreate full maturation and functionality of neutrophils and platelets and in part red blood cells.

## Data Availability Statement

The original contributions presented in the study are included in the article/supplementary material. Further inquiries can be directed to the corresponding authors.

## Author Contributions

WL and PT contributed equally to generating the first draft of the manuscript. SH and GG contributed equally to designing and coordinating the completion of this project. All authors read and approved the final version of the manuscript.

## Funding

GG was supported by the following grants: R01 CA253981, 1P01CA225618, ASH Bridge award and P30 CA006973-57S2. SH was supported by the following grants: R01 CA253981, R01DK102792, The Frederick A. DeLuca Foundation, Vera and Joseph Dresner Foundation, and The U.S. Department of Defense Peer Reviewed Cancer Research Program, Expansion Award (CA171025, W81XWH1810138).

## Conflict of Interest

The authors declare that the research was conducted in the absence of any commercial or financial relationships that could be construed as a potential conflict of interest.

## Publisher’s Note

All claims expressed in this article are solely those of the authors and do not necessarily represent those of their affiliated organizations, or those of the publisher, the editors and the reviewers. Any product that may be evaluated in this article, or claim that may be made by its manufacturer, is not guaranteed or endorsed by the publisher.
